# New Mononuclear and Binuclear Cu(II), Co(II), Ni(II), and Zn(II) Thiosemicarbazone Complexes with Potential Biological Activity: Antimicrobial and Molecular Docking Study

**DOI:** 10.3390/molecules26082288

**Published:** 2021-04-15

**Authors:** Ahmed Gaber, Moamen S. Refat, Arafa A.M. Belal, Ibrahim M. El-Deen, Nader Hassan, Rozan Zakaria, Majid Alhomrani, Abdulhakeem S. Alamri, Walaa F. Alsanie, Essa M. Saied

**Affiliations:** 1 Department of Biology, College of Science, Taif University, P.O. Box 11099, Taif 21944, Saudi Arabia; a.gaber@tu.edu.sa; 2 Center of Biomedical Sciences Research, Taif University, P.O. Box 11099, Taif 21944, Saudi Arabia; m.alhomrani@tu.edu.sa (M.A.); a.alamri@tu.edu.sa (A.S.A.); w.alsanie@tu.edu.sa (W.F.A.); 3 Department of Chemistry, College of Science, Taif University, P.O. Box 11099, Taif 21944, Saudi Arabia; 4 Department of Chemistry, Faculty of Science, Port Said University, Port Said 42511, Egypt; a.belal@psu.edu.eg (A.A.M.B.); i.eldeen@psu.edu.eg (I.M.E.-D.); n.hassan@psu.edu.eg (N.H.); rozan.zakaria@yahoo.com (R.Z.); 5 Department of Clinical Laboratory Sciences, College of Applied Medical Sciences, Taif University, P.O. Box 11099, Taif 21944, Saudi Arabia; 6 Chemistry Department, Faculty of Science, Suez Canal University, P.O. Box 41522, Ismailia 42524, Egypt; Saiedess@hu-berlin.de; 7 Institute for Chemistry, Humboldt Universität zu Berlin, Brook-Taylor-Str. 2, 12489 Berlin, Germany

**Keywords:** methoxy thiosemicarbazone, metallodrugs, transition metals, metal complexes, antimicrobial, molecular docking

## Abstract

Herein, we report the synthesis of eight new mononuclear and binuclear Co^2+^, Ni^2+^, Cu^2+^, and Zn^2+^ methoxy thiosemicarbazone (MTSC) complexes aiming at obtaining thiosemicarbazone complex with potent biological activity. The structure of the MTSC ligand and its metal complexes was fully characterized by elemental analysis, spectroscopic techniques (NMR, FTIR, UV-Vis), molar conductivity, thermogravimetric analysis (TG), and thermal differential analysis (DrTGA). The spectral and analytical data revealed that the obtained thiosemicarbazone-metal complexes have octahedral geometry around the metal center, except for the Zn^2+^-thiosemicarbazone complexes, which showed a tetrahedral geometry. The antibacterial and antifungal activities of the MTSC ligand and its (Co^2+^, Ni^2+^, Cu^2+^, and Zn^2+^) metal complexes were also investigated. Interestingly, the antibacterial activity of MTSC- metal complexes against examined bacteria was higher than that of the MTSC alone, which indicates that metal complexation improved the antibacterial activity of the parent ligand. Among different metal complexes, the MTSC- mono- and binuclear Cu^2+^ complexes showed significant antibacterial activity against *Bacillus subtilis* and *Proteus vulgaris*, better than that of the standard gentamycin drug. The in silico molecular docking study has revealed that the MTSC ligand could be a potential inhibitor for the oxidoreductase protein.

## 1. Introduction

Infectious diseases caused by various bacterial fungal and viral pathogens are of high socioeconomic and medical importance worldwide. Among the infectious diseases that are very challenging to treat, biofilm-associated pathogens are one of the most dangerous. The exclusive physiology and intricate structure of the biofilm cells contribute to their resistance to host immune response, environmental conditions, and antimicrobial agents [1]. As a standard therapy, these infections are commonly treated with broad-spectrum antibiotics. However, these broad-spectrum antibiotics have side effects, such as affecting the normal microbial flora and inducing antibiotic resistance [2]. Therefore, the discovery of new antimicrobial drugs with a novel mode of action is urgently needed. Potentially, these alternative strategies may also lead to the discovery of novel pharmacological targets, which would also reduce the application of broad-spectrum antibiotics in clinics and thereby reduce the emergence of antibiotic resistance to these important antibiotics.

**Scheme 1:**
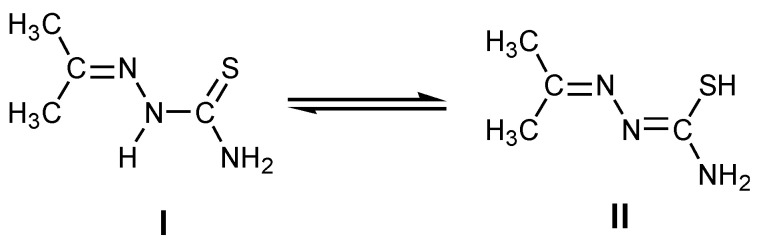
Structures of thiosemicarbazone in thione-thiol tautomerism.

Thiosemicarbazone derivatives and their metal complexes have been extensively explored in pharmacology due to their broad spectrum of biological activities, including antimicrobial, anticancer, and antiviral activities [3,4,5,6,7,8,9,10,11]. Thiosemicarbazones are known to have a general formula of R_1_R_2_C=N-NH-C=S-NR_3_R_4,_ which make them significant donor ligands through their hydrazine nitrogen and sulfur atoms. They can also coordinate as multi-dentate ligands depending on the presence of other heteroatoms, which can act as donating centers to form complexes with different coordination geometries. Thiosemicarbazones can coordinate as bidentate ligands with the central metal ion as neutral thione form or anionic thiolate form to provide a five-membered chelating ring (Scheme 1) [12].

They display as thione-thiol tautomerism due to the existence of the –NH-C=S functional group. The thione form I of thiosemicarbazone was found in the solid state, while in the liquid state, it tautomerizes to the thiol form II [13,14]. In alkaline solution, structure II is more favored [15]. This class of compounds has attracted more attention after the discovery of the first thiosemicarbazone-based drug, 2-formylpyridine thiosemicarbazone, which showed a potential anticancer activity [16]. Over the last decades, extensive research has successfully led to the discovery of many thiosemicarbazone-based drugs. For example, the methisazone (Marboran^®^) as antiviral to treat smallpox [17], 3-aminopyridine-2-carboxaldehyde thiosemicarbazone (Triapine^®^) as an antitumor drug [18,19], and di-2-pyridylketone 4-cyclohexyl-4-methyl-3-thiosemicarbazone (DpC) and (E)-N′-(6,7-dihydroquinolin-8-(5H)-ylidene)-4-(pyridine-2-yl)piperazine-1 carbothiohydrazide (COTI-2), which recently entered clinical trials [20,21]. Interestingly, since thiosemicarbazones have an excellent ability to chelate with a wide range of biologically relevant metal ions, it was found that their mode of action always involves their interaction with metal ions [22]. The complexation of these multi-dentate thiosemicarbazones to the metal ions might result in metal complexes with enhanced biological activities and altered their modes of action [23,24,25,26,27,28].

Based on these facts and in continuing our efforts in the synthesis of bioactive metal complexes [29,30,31], herein we report the synthesis of new methoxy thiosemicarbazone derivative (MTSC) starting from 3-methoxy-2-hydroxybenzaldehyde. The synthesized thiosemicarbazone derivative was used as a multi-dentate ligand to synthesize a set of eight new metal complexes. The structure of synthesized metal complexes was fully characterized by a broad spectrum of analysis. The biological activity of the MTSC and its metal complexes was also examined using various microorganisms. Furthermore, we have performed a molecular docking study for the MTSC ligand to validate its biological activity.

## 2. Materials and Methods

### 2.1. General Description of Materials and Methods

All commercially available chemical reagents and solvents were purchased from Sigma-Aldrich or Merck and used without further purification unless otherwise specified. All the solvents were used after distillation by standard methods. The metal contents were determined gravimetrically by converting the metals into their corresponding oxides. Chloride ions were also determined gravimetrically to confirm the proposed structure for the complexes. Elemental analyses (carbon, hydrogen, and nitrogen content) were performed using VARIO EL III GERMANY elemental analyzer in the Microanalytical Center of Cairo University. The thermogravimetric analysis (TGA) and differential thermogravimetric (DTG) were performed under nitrogen atmosphere using a Shimadzu thermogravimetric analyzer with a heating rate of 10 °C min^−1^ up to 800 °C. Molar conductivities of a freshly prepared solution (10^−3^ mol/dm^3^) in dimethylformamide (DMF) were conducted using Jenway 4010 conductivity meter. Magnetic measurements were carried out using a Sherwood Scientific Cambridge balance. ^1^H-NMR spectra (400 MHz) were recorded by Varian Gemini spectrophotometers. IR analysis (4000–400 cm^−1^) was performed using a Bruker FT-IR spectrophotometer at Cairo University. Mass spectra were conducted at room temperature using mass spectrometer AEI MS 30 at 70 eV.

### 2.2. Synthetic Procedures and Analytic Data of Compounds

#### 2.2.1. (E)-2-(2-hydroxy-3-methoxybenzylidene)hydrazine-1-carbothioamide Synthesis (**3**)

A solution of thiosemicarbazide (0.1 mmol) and 3-methoxy-2-hydroxybenzaldehyde (0.1 mmol) in ethanol (50 mL, 0.01 M) was allowed to reflux for 4 h. The obtained precipitate was filtered, washed with ethanol, and dried. The obtained residue was recrystallized with ethanol to produce compound **3**. M.P = 280 °C; IR (KBr) = 3398–2980 cm^−1^ (OH), 3129 cm^−1^ (NH_2_), 3283 cm^−1^ (NH), 1620 cm^−1^ (CH=N), 1598 and 1565 cm^−1^ (C=C), 1070 and 1028 cm^−1^ (C-O); ^1^HNMR (DMSO-d_6_, ppm) δ: 3.85 (s,3H,OCH_3_), 6.98 (s, 2H, NH_2_), 7.08–7.49 (m,4H,Ar-H), 8.60 (s,1H,CH=N), 9.78 (br.s,1H,OH), 10.67 (s,1H,NH).

#### 2.2.2. Ethyl-3-(4-methoxyphenyl-2-cyanoacrylate) Synthesis (**6**)

A solution of 4-methoxy benzaldehyde (1 mmol) and ethylcyanoacetate (1.5 mmol) in ethanol (50 mL, 0.01 M) was added with piperidine (1 mL). The resulting reaction mixture was allowed to reflux for 2 h. Subsequently, the resulted mixture was cooled to ambient temperature and was then poured into ice-cold water. The obtained mixture was neutralized with dilute hydrochloric acid (20%), leading to the formation of a solid precipitate. The solid precipitate was separated, washed, dried, and recrystallized using ethanol to produce ethyl-3-(p-methoxyphenyl)-2-cyanoacrylate **6**. M.P = 88 °C; IR(KBr) = 2230 cm^−1^ (CN), 1731 cm^−1^ (C=O), 1613 and 1595 cm^−1^ (C=C), 1117, 1083, and 1017 cm^−1^ (C-O); ^1^HNMR (DMSO-d_6_) δ: 1.37 (t, 3H, CH_3_), 3.88 (s, 3H, OCH_3_), 4.30 (q, 2H, OCH_2_), 7.15 (d, 2H, Ar-H), 8.09 (d, 2H, Ar-H), 8.30 (s, 1H, H-olefenic) ppm.

#### 2.2.3. Ethyl (E)-2-Cyano-3-(2-((E)-2-hydroxy-3-methoxybenzylidene) Hydrazine-1-carbothioamido)-3-(4-methoxyhenyl) Acrylate (MTSC Ligand) Synthesis (**7**)

A mixture of thiosemicarbazone derivative (**3**) (1 mmol) and ethyl cyanoacetate (1 mmol) in ethanol (50 mL) was treated with fused sodium acetate (3 mmol) and the resultant reaction mixture was allowed to reflux for 4 h. The reaction mixture was left to cool down and poured into water. The desired precipitate was filtered, dried and recrystallized with ethanol to afford MTSC ligand **7**. M.P = 210 °C; IR(KBr) = 3029 cm^−1^ (CH=N), 2214 cm^−1^ (CN), 1716 cm^−1^ (C=O), 1558 and 1512 cm^−1^ (C=C), 1589 cm^−1^ (C=N), 1211 and 1091 cm^−1^ (C-O); ^1^HNMR (DMSO-d_6_) δ: 1.29 (t,3H,CH_3_), 3.81 (s,3H,OCH_3_), 3.84 (s,3H,OCH3), 4.30 (q,2H,OCH_2_), 6.74–8.14 (m,7H,Ar-H), 7.91 (s,1H,NH) ppm, 9.20 (s,1H,CH=N), 11.43 (s,1H,OH); MS *m*/*z* found: 454, calc. for: C_22_H_22_N_4_O_5_S: 454 (M^+^).

#### 2.2.4. Synthesis of MTSC-Metal Complexes

(1:1) Metal Complexes Synthesis

A hot solution of MTSC ligand **7** (1 mmol) in methanol (30 mL) was treated drop-wisely with a hot solution of metal (II) chloride MCl_2_ (1 mmol) (CoCl_2_.6H_2_O, NiCl_2_.6H_2_O, CuCl_2_.2H_2_O, and ZnCl_2_) in methanol (20 mL) added to another. The resulting reaction mixture was stirred under reflux at 70 °C for 1 h. Subsequently, the pH of the reaction mixture was adjusted to pH 8–9 by adding drops of ammonia solution, and then the solvent was evaporated to half of its preliminary volume. The resulting mixture was allowed to set in the fridge overnight, while a solid precipitate was formed. The obtained metal complex was filtered, dried with vacuum by anhydrous calcium chloride, and washed with methanol. The final metal complex was obtained in a pure form after recrystallization with diethyl ether.

^1^H-NMR spectrum of (1:1) Zn(II) complex (DMSO-d_6_) δ: 1.24 (t,3H,CH_3_), 3.37 (s,3H,OCH_3_), 3.70 (s,3H,OCH_3_), 3.86 (q,2H,OCH_2_), 6.35–8.11 (m,7H,Ar-H), and 8.34 (s,1H,CH=N).

(1:2) Metal Complexes Synthesis

A procedure was followed as described above. However, in this case, 2 mmol of the metal (II) chloride was used to react with MTSC ligand **7** (1 mmol).

^1^H-NMR spectrum of (1:2) Zn(II) complex ((DMSO-d_6_) δ: 1.30 (t,3H,CH_3_), 3.71 (s,3H,OCH_3_), 3.85 (s,3H,OCH3), 3.88 (q,2H,OCH_2_), 6.17–8.20 (m,7H,Ar-H) and 8.33 (s,1H,CH=N)).

### 2.3. Determination of the Antibacterial and Antifungal Activities

The antibacterial and antifungal activities of the MTSC ligand and its metal complexes were evaluated following standard protocols. The antibacterial activity was examined against the Gram-positive bacteria (*Bacillus subtilis* and *Staphylococcus aureus*) and the Gram-negative bacteria (*E. coli* and *Proteus vulgaris*). Agar well diffusion method was used to determine the antibacterial activity [32]. On the surface of nutrient agar (pH 6.6 to 7.0), centrifuged granules were widespread after it was autoclaved for 20 min at 121 °C. A sample of the tested compound (10 µg/mL) was prepared in DMSO. The standard drug gentamicin was used as a standard antibacterial drug at a concentration of 1.2 μg/mL. The inhibition zone was measured 3 times to determine the activity of the samples, and the average was taken. On the other hand, the antifungal activity was examined against *Aspergillus flavus* and *Candida albicans*. Briefly, the fungal plates were prepared by growing the fungus in 5 mL of sabouraud dextrose broth until the concentration of the cells reached 105 CFU/mL. A stock solution of the tested compound was prepared in DMSO (10 µg/mL). Ketoconazole was used as a standard antifungal drug [33].

### 2.4. Molecular Docking Study

The bonding affinity of MTSC ligand (**7**) to 3hb5-oxidoreductase protein and speckle-type POZ protein (SPOP) protein binding was investigated by in silico molecular docking. The molecular modeling was performed using Docking Server [34]. The energy of the 3D ligand was minimized by the MMFF94 force field [35]. Some parameters were added to aid the process of AutoDocking as hydrogen atoms, Kollman united atom type charges, and solvation parameters [36]. Van der Waals and the electrical terms were calculated. Lamarckian genetic algorithm (LGA) and the Solis and Wets local search method were used for the AutoDocking program [37].

### 2.5. Statistical Analysis

Statistical comparisons were measured by a one-way ANOVA with the Duncan test using IBM SPSS version 26. A probability level of 0.05 or lower was considered statistically significant.

## 3. Results and Discussion

### 3.1. Synthesis of MTSC Ligand

**Figure 1:**
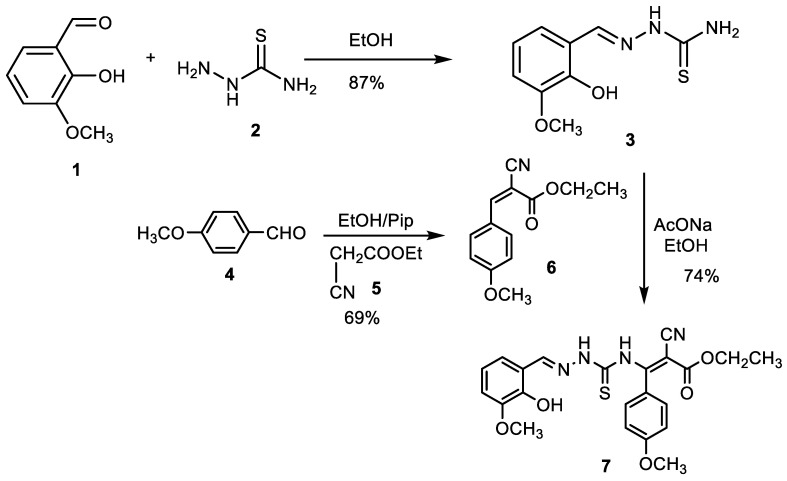
Reagents and conditions for the synthesis of the methoxy thiosemicarbazone (MTSC) ligand (**7**).

The synthesis of MTSC ligand (**7**) was started by the condensation of 3-methoxy-2-hydroxybenzaldehyde (**1**) with thiosemicarbazide to afford the thiosemicarbazone derivatives (**3**) (Figure 1). Condensation of 4-methoxybenzaldehyde (**4**) with ethyl cyanoacetate in the presence of a catalytic amount of piperidine afforded the ethyl-3-(p-methoxyphenyl)-2-cyanoacrylate (**6**) in a 69% yield. Finally, thiosemicarbazone derivative (**3**) was reacted with ethyl 3-(4-methoxyphenyl)-2-cyanoacrylate (**6**) in the presence of fused sodium acetate to furnish the desired methoxy thiosemicarbazone ligand (**7**) in a 74% yield.

### 3.2. Elemental Analyses and Physical Properties

The physical and chemical properties of MTSC-metal complexes are summarized in Table 1. The molar conductivity measurements for metal complexes showed very low values (5–11) μS compared to the free ligand (29 μS), which indicates that the synthesized metal complexes are non-electrolytic [38]. The metal complexes were non-crystalline, non-hygroscopic, and colored solid with high stability as they depredated above 250 °C. As expected, all complexes were only soluble in DMF or DMSO. The ratio between metal and ligand was determined by elemental (carbon, hydrogen, nitrogen, sulfur, chloride, and metal ions content) and thermogravimetric analyses. The melting point of all complexes is above 300 °C, and the yield of isolated solid metal complexes is inserted within 72–79%.

### 3.3. Infrared Spectral Studies

The spectral bands of the MTSC ligand and its metal complexes are briefly summarized and illustrated in Table 2. All the metal complexes showed a wide range of different intensity bands. The IR spectra of the MTSC free ligand revealed the peak of the azomethine group (CH=N) at 1589 cm^−1^. This peak was shifted to lower wavenumbers in the spectra of the metal complexes at scale range of 1504–1573 and 1508–1527 cm^−1^ in (1:1) and (1:2) complexes, respectively. This indicates that the (CH=N) group is contributed to the complexation process through its nitrogen atom. At 2214 cm^−1^, a band for *υ*(C≡N) appeared in the spectrum of the MTSC ligand. This band was not found in the metal complexes, which indicates their hydrolysis during complexation reaction. Broadband at 3340 cm^−1^ for *υ*(OH) was noticed in the spectrum of the MTSC ligand [38], which disappeared in the metal complexes, which demonstrates the role of the OH group in the complexation process. The wideband found at scale 3309–3344 cm^−1^ in the spectra of the (1:1) metal complexes and also at range 3317–3336 cm^−1^ in (1:2) metal complexes revealed the vibration of *υ*(H_2_O) [39]. This band gives great evidence for the contribution of water molecules in complex formation. At 1211 cm^−1,^ an average band appeared in the spectra of the MTSC ligand, which corresponds to the phenolic oxygen *υ*(C-O). After complexation, the band was shifted to a lower frequency, which indicated metal-oxygen bond formation. The *υ_as_*COO (antisymmetric) and *υ_s_*COO (symmetric) frequencies of the carboxylic ions appeared in the range of 1384–1562 and 1589–1612 cm^−1^. The difference between the stretching vibration motions demonstrated that the carboxylate groups act as monodentate when coordinating with the metal ions. The bands of *ν*(C=S) and ν(COCH_3_) (1018 cm^−1^, 2843 cm^−1^_,_ respectively) in the spectra of free ligand did not show any shift after complexation, which indicates that C=S and COCH_3_ groups are not contributing in the complexation with the metal ion. In the spectra of (1:1) metal complexes, the stretching vibration frequency of carboxylic group *υ*(C=O) pointed at 1713–1722 cm^−1^ [39]. This value was shifted in (1:2) complexes which could attribute to the involvement of the (C=O) group in the coordination with the central metal ion. New bands appeared at 3116–3194 and 3012–3232 cm^−1^ in spectra of (1:1) and (1:2) metal complexes consecutively, which referred to *υ*(NH) of the -NH_3_ group. This indicates that the nitrogen atom plays a role in the coordination with the metal ion. Additionally, new bands appeared at 516–597 and 416–495 cm^−1^ in the metal complexes spectra, which could be referred to as the M-O and M-N binding [39].

### 3.4. UV-Vis and Magnetic Studies

The magnetic measurements were performed following Guoy’s method [40]. The UV-Vis absorption was measured for a solution of MTSC and its metal complexes in DMSO. The absorption spectra of the MTSC ligand showed a band at 380 nm, which refers to the *n*-π* transition of the azomethine group. After metal coordination, this band was shifted to a higher value (395–400 nm), which indicates the contribution of the azomethine group through its nitrogen atom toward the coordination to the central metal ion. The transitions at 330 nm indicated the π-π* transitions of the aromatic rings in the MTSC ligand. These bands were also shifted in the metal complex spectrum to higher wavelength values (~340 nm). The Co(II) complexes showed magnetic moment values of 4.17–4.32 BM, which confirm the presence of three unpaired electrons and indicate the octahedral geometry of the Co(II) complexes. The UV-Vis spectrum of the Co(II) complexes showed bands with frequencies at ranges 910–900, 552–550, and 432–430 nm, which could be attributed to the following: ^4^T_1g_(F)*→*^4^T_2g_ (*ν_1_*), ^4^T_1g_*→*^4^A_2g_ (*ν*_2_), and ^4^T_1g_(F)*→*^4^T_1g_ (P) (*ν*_3_), consecutively [41]. The Ni(II) complexes stated magnetic moment values at a scale of 3.14–3.35 BM with high spin, which indicates the octahedral geometry of Ni(II) complexes [42]. The electronic spectrum of the Ni(II) complexes showed three major bands at 875–865, 625–612, and 400–395 nm, which could be implied to ^3^A_2__g_→^3^T_2__g_ (F) (*ν*_1_), ^3^A_2__g_→^3^T_1__g_(F) (*ν*_2_) and ^3^A_2__g_→^3^T_2__g_ (F) (*ν*_3_) transitions, consecutively. The Cu(II) complexes recorded magnetic values at a range of 1.24–1.31 BM. The UV-Vis electronic spectra of Cu(II) complexes recorded absorption values at 860–850 nm, 560–550 nm, and 426–420 nm, which could be contributed to the ^2^B_1g_→^2^A_1g_, ^2^B_1g_→^2^B_2g_, and ^2^B→^2^E_g_ [42,43] transitions consecutively. The diamagnetic properties of Zn(II) complexes were confirmed by the absence of d–d frequencies and domination of frequencies at ~325 nm for *n*/π-π* transition [42].

### 3.5. ^1^H-NMR Spectra Studies

The ^1^H-NMR analysis provided evidence for the structures of the MTSC-metal complexes. In the ^1^H-NMR spectrum of MTSC free ligand (Figure S1), the distinguish peaks for OH, HC=N, and NH protons were found at δ = 11.43, 9.19, and 7.90 ppm, respectively. The distinguish peak of the OH group was disappeared in Zn (II) complexes which indicates the contribution of the OH group in the coordination with the metal ion (Figure S2). The distinguish proton of the HC=N group was shifted upon Zn (II) complexation to a lower value of 8.33 ppm, which indicates the involvement of the HC=N group in the complexation process. The proton of the NH group was presented in the spectra of the Zn (II) complexes without any shift, which implies the absence of complexation of the NH group to the metal ion. In spectra of the mononuclear Zn (II) complex, a new peak appeared at 9.20 ppm, which was assigned for the COOH group (Figure S3). Noteworthy, this proton peak was disappeared in the binuclear Zn (II) complex, which indicates the contribution of the COOH group to the coordination of the second metal ion. In the ligand spectra, the set of peaks at 6.74–7.50 and 8.06–8.41 ppm was assigned to the aromatic protons. These peaks were presented in the same range in the Zn (II) complexes (6.34–7.15 and 8.09–8.11), indicating that the aromatic protons are not contributing in the coordination to the metal ion. The peaks that appeared at 2.50 and 3.70 ppm in (1:1) and (1:2) Zn (II) complexes were referring to DMSO and H_2_O/DMSO.

### 3.6. Thermo Gravimetric Studies

The thermogravimetric (TG) analysis for the MTSC and its metal complexes was performed under nitrogen conditions with 10 °C/min heating rate. The steps for the TG decomposition of the MTSC ligand and its metal complexes are summarized in Table 3. The thermal degradation of the free ligand and its metal complexes is illustrated and by the percentage of mass loss through temperature periods.

#### 3.6.1. MTSC Ligand

From the TG curve, the MTSC ligand was degraded in three main steps within the range of 20–800 °C. The first degradation step occurred with a weight loss of 69% (calc. = 69.7%) within a temperature range of 20–295 °C. In the second degradation step, the weight loss was 21% (calc. = 20%) within the temperature range of 295–530 °C. Residual up to 800 °C was escorted with a weight loss of 10% (calc. = 10.1%).

#### 3.6.2. MTSC-Metal Complexes in 1:1 Ratio (Metal:Ligand)

Co (II) Complex 8

The thermal analysis of the Co(II) complex showed a strong DTG_max_ degradation stage (Figure S4). The first decomposition stage occurred within a temperature range of 35–203 °C with mass loss of 8% (calc. = 7.8%), which coincided with the degradation of three uncoordinated water molecules. The second stage involved the weight loss of 52% (calc. = 52.5%) within a temperature range of 203–460 °C, which referred to the loss of three water molecules, hydrogen, ammonia, hydrogen sulfide, nitrogen, chlorine, and acetylene gas molecules. Continuous degradation occurred with weight loss of 13% (calc. = 13.1%) at a temperature range of 460–620 °C, which indicated the loss of carbon dioxide and nitrogen oxide gas. The residue of pure metal and the eventual product were stable up to 800 °C. Co metal production was performed by carbothermal reduction where CoO produced from the degradation of cobalt carbonate was conversed as Co pure metal.

Ni (II) Complex 10

The structural formula of the Ni(II) complex was proposed from the TG and differential thermal analysis (Figure S5). The TGA curve indicated that the nickel (II) complex degraded in three principal decomposition stages. In the first stage, the weight loss was 5% (calc. = 5.5%) with a DTG_max_ of 45 °C within a temperature range of 35–249 °C. The mass loss coincided with the loss of two water molecules. The second thermal degradation step occurred within a temperature range of 249–370 °C and involved the loss of water molecule, ammonia, chloride, hydrogen sulfide, and hydrogen gas molecules with DTG_max_ = 300 °C (obs. = 23%, calc. = 22.4%). In the third step, the weight loss was 40.5% (calc. = 40.5%) which referred to the loss of water molecule, nitrogen, nitrogen oxide, carbon dioxide, and acetylene gas molecules with DTG_max_ = 445 °C (temperature range 370–585 °C). Finally, the NiO and the remaining residue of organic moiety (residual carbon) were stable until 800 °C.

Copper (II) Complex 12

The thermal decomposition Cu(II) complex occurred mainly in four decomposition stages with DTG_max_ of 90 (20–175 °C), 210, 255, 330 (175–405 °C), 504 (405–605 °C), and 650 (605–710 °C) (Figure S6). The first phase of degradation involved the loss of three water molecules (obs. = 8, calc. = 8) with DTG_max_ = 90 °C. The second, third, and fourth degradation phases occurred at DTG_max_ of 210, 255, 330, 504, and 650 °C. The weight loss recorded at these phases was corresponding to two water molecules, ethane, hydrogen sulfide, ammonia, nitrogen oxide, carbon dioxide, and chlorine gas (obs. = 61%, calc. = 60.8%). The residues were the CuO and the residual organic moiety.

Zinc (II) Complex 14

The thermal decomposition of the Zn(II) complex occurred in three main decomposition steps with DTG_max_ of 120, 140 (30–180 °C), 255 (180–450 °C), and 530, 570 (450–630 °C) (Figure S7). The first thermal decomposition step occurred within a temperature range of 30–180 °C and involved the loss of water molecule (obs. = 3%, calc. = 2.9%). Then, water molecule, nitrogen oxide, and acetylene gas molecules (obs. = 35%, calc. = 35.1%) were removed in the second stage with DTG_max_ of 255 °C (temperature range 180–450 °C). The third step occurred within a temperature range of 450–630 °C and included the loss of water molecule, nitrogen, carbon dioxide, chlorine, ammonia, and hydrogen gas molecules (obs. = 30%, calc.= 32%). Organic residue and zinc metal were obtained at 630 °C.

#### 3.6.3. MTSC-Metal Complexes in 2:1 Ratio (Metal:Ligand)

Cobalt (II) Complex 9

The TG analysis of the Co(II) complex recorded strong DTG_max_ degradation stages (Figure S4). The first decomposition phase occurred within the temperature range of 35–180 °C with a mass loss of 13%, which referred to the loss of uncoordinated water molecules. In the second degradation step, the mass loss was 52% (calc. = 51.7%), which coincided with the loss of ammonia, acetylene, carbon dioxide, hydrogen sulfide, and hydrogen gas molecules within the temperature range 180–420 °C. The third degradation step involved the loss of water, ammonia, nitrogen, and chlorine gas molecules within a temperature range of 420–547 °C. The residues of pure metal and organic moiety were stable until 800 °C. Co metal production was conducted by carbothermal reduction where CoO produced from the degradation of cobalt carbonate was conversed as pure Co metal.

Nickel (II) Complex 11

The nickel (II) complex preceded its thermal degradation mainly in three decomposition steps (Figure S5). First, two water molecules and ammonia gas (obs. = 11, calc. = 10.9%) were removed within a temperature range of 25–230 °C with a DTG_max_ of 145 °C. The second stage occurred within a temperature range of 230–375 °C and involved the mass loss of 19% (calc. = 18.5%), which coincided with the loss of ammonia, hydrogen sulfide, chlorine, and hydrogen gas molecules. In the third step, the weight loss involved the loss of water, acetylene, nitrogen oxide, and nitrogen gas molecules (obs. = 34%, calc. = 33.9%) within a temperature range of 375–551 °C. The remaining organic residues and NiO were stable until 800 °C.

Copper (II) Complex 13

The thermal degradation of the Cu(II) complex occurred basically in three degradation stages with DTG_max_ of 40 (20–155 °C), 215, 305, 359 (155–525 °C), and 655 (525–740 °C) (Figure S6). In first phase of degradation, the water molecules (obs. = 4%, calc. = 4.4%) were removed with DTG_max_ of 40 °C. The second step occurred within the temperature range of 155–525 °C and involved the loss of ammonia, hydrogen sulfide, chlorine, and hydrogen gas molecules (obs. = 38, calc. = 37.8%) with a DTG_max_ of 215, 305, 359 °C. The third decomposition phase implicated to the loss of nitrogen oxide gas (obs. = 6%, calc. = 5.7%) with DTG_max_ of 655 °C (temperature range 525–740 °C). The remaining residues were the CuO and the organic residues.

Zinc(II) Complex 15

The Zn(II) complex degradation occurred in two main decomposition steps with DTG_max_ of 160, 245 (25–345 °C) and 540 (35–545 °C) (Figure S7). The first phase involved the loss of uncoordinated water molecule (obs. = 46%, calc. = 45.5%) with DTG_max_ of 160 °C. Then, water molecule, chlorine, ammonia, hydrogen sulfide, and methane gas molecules (obs. = 21%, calc. = 21.4%) were removed in the second stage with DTG_max_ of 540 (temperature range 345–545 °C). Carbon residues and zinc metal were obtained at 545 °C.

### 3.7. Kinetic Thermodynamic Parameters

Next, it was important to support the thermal degradation studies for the MTSC ligand and its metal complexes with kinetic thermodynamic calculations. These analyses provided adequate information on Arrhenius parameters viz. activation energy (E*), frequency factor (A), enthalpy of activation (H*), the entropy of activation (S*), and free energy of activation (G*). The thermodynamic kinetic parameters have been computed and evaluated by TG/DTG curves following the Coats–Redfern (CR) and Horowitz–Metzger (HM) methods [44,45]. Analysis of TG curves provides a convenient approach for the determination of the rate-dependent parameters of non-isothermal degradation reactions. Serval studies reported the advantages of analyzing TG curves over the conventional isothermal protocols to obtain the values of thermodynamic parameters [46,47,48,49,50,51]. The thermodynamic data are summarized in Table 4. The thermodynamic data obtained from the CR and HM methods were inadequate with each other. The computed values of E*, A, and S* referred to the rate of the reaction for the starting reactants and intermolecular compounds. The increased values of activation energy indicated the high stability of the metal complexes. The negative values of S* revealed that the metal complexes are more highly ordered than the ligands. As detailed in Table 1, the Arrhenius plots of the thermal degradation steps showed correlation coefficient values of 0.90–0.99, which indicated the fit with the linear function. Additionally, the thermodynamic data showed that the thermal decomposition of all MTSC-metal complexes is non-spontaneous, which indicates that all MTSC-metal complexes are thermally stable.

### 3.8. Proposed Structure of Synthesized Complexes

**Figure 2:**
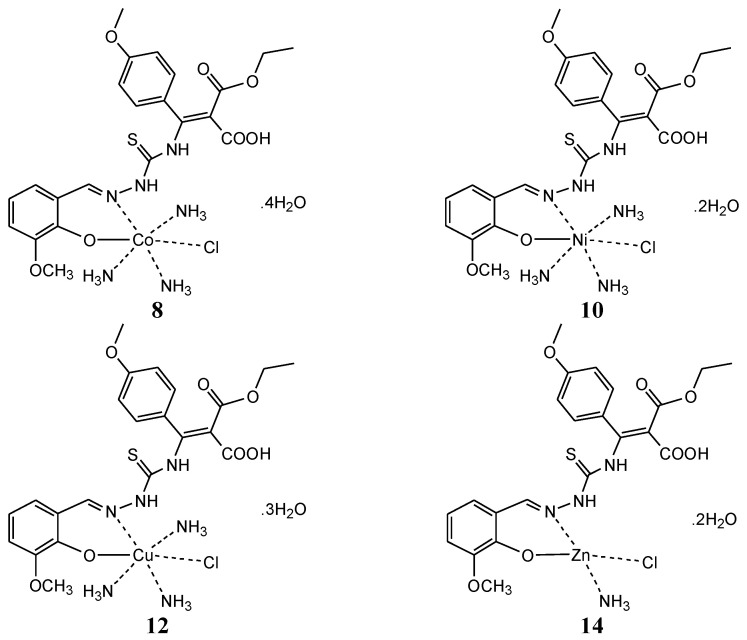
Proposed structures of 1:1 MTSC complexes (**8**, **10**, **12**, **14**).

**Figure 3:**
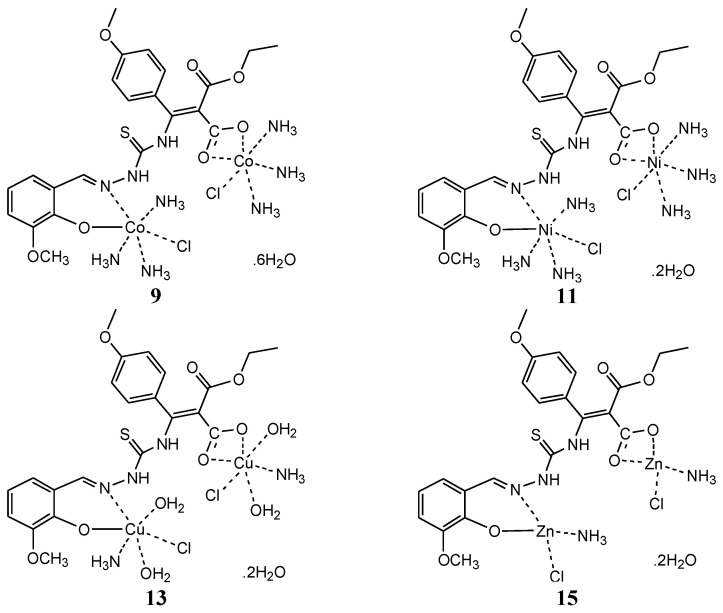
Proposed structures of 1:2 MTSC complexes (**9**, **11**, **13**, **15**).

The synthesized metal complexes were obtained as a powder, not as a single crystal; therefore, the exact structure of obtained metal complexes cannot be fully characterized by X-ray studies. Nevertheless, based on the wide spectrum of analytical analysis that has been performed, such as elemental analysis, ^1^H-NMR, FTIR analysis, UV-Vis, molar conductivity, thermographic analysis as well as magnetic measurements, the obtained thiosemicarbazone-metal complexes have octahedral geometry around the metal center, except for the Zn^2+^-thiosemicarbazone complexes, which showed a tetrahedral geometry. In the case of mononuclear MTSC-metal complexes, the MTSC ligand is coordinated to the metal (II) chloride as a bi-dentate ligand through the lone pairs of the phenolic oxygen and the azomethine nitrogen (Figure 2). However, in the case of binuclear MTSC-metal complexes, the second metal (II) chloride is coordinated with the carboxylic oxygen atoms (Figure 3). The ratio between metal and ligand (metal:ligand, 1:1 and 2:1) was proposed based on the elemental analysis and thermogravimetric analyses. The suggested molecular formulas of the MTSC-metal complexes as follows: [Co(MTSC)(NH_3_)_2_(Cl)(H_2_O)].6H_2_O (**8**), [Co_2_(MTSC)(NH_3_)_2_ (Cl)_2_(H_2_O)_4_].2H_2_O (**9**), [Ni(MTSC)(NH_3_)_3_(Cl)].2H_2_O (**10**), [Ni_2_(MTSC)(NH_3_)_4_(Cl)_2_ (H_2_O)_2_].4H_2_O (**11**), [Cu(MTSC)(NH_3_)_3_(Cl)].2H_2_O (**12**), [Cu_2_(MTSC)(NH_3_)_4_(Cl)_2_(H_2_O)_2_].2H_2_O (**13**), [Zn(MTSC)(NH_3_)(Cl)].3H_2_O (**14**), and [Zn_2_(MTSC)(NH_3_)_3_(Cl)_2_].2H_2_O (**15**).

### 3.9. Biological Activity Evaluation

#### 3.9.1. Antibacterial Assessments of MTSC Ligand and Its Metal Complexes

The antibacterial activity of the MTSC ligand and its metal complexes is summarized in Table 5. The hole diffusion protocol was employed, and the activity of the compounds was examined against Gram-positive bacteria (*Bacillus subtilis* and *Staphylococcus aureus*) and Gram-negative bacteria (*E. coli* and *Proteus vulgaris*). The activity of tested compounds was assessed at a concentration of 1.0 µM and compared to that of the control (DMSO and gentamycin). From literature, 0.1 and 1 µM of Cu(II), Ni(II), and Zn(II) complexes with salicylidene thiosemicarbazones is not a cytotoxic concentration [52].

As detailed in Table 5, the MTSC-metal complex showed a significantly higher antibacterial activity than the MTSC ligand (Table 5). The MTSC ligand showed moderate antibacterial activity against most of the tested bacteria. Interestingly, as previously reported [53], the activity of the MTSC ligand was significantly improved upon metal complexation. All MTSC-metal complexes showed considerable broad-spectrum antibacterial activities against both Gram-positive and Gram-negative bacteria. Both mononuclear and binuclear MTSC complexes showed similar activities, which indicates that the second metal coordination has no considerable effect on the antibacterial activity of the complexes. Among tested MTSC-metal complexes, the mononuclear and binuclear Cu (II)-MTSC complexes showed to be the most active metal complexes against examined bacteria. Noteworthy, the antibacterial activity of Cu(II)-MTSC complexes against *Bacillus subtilis* and *Proteus vulgaris* bacteria was higher than that of the antibacterial drug gentamycin. These novel findings indicate that the Cu(II)-MTSC complexes are highly effective antibacterial agents. Further studies should be directed in the future to investigate the broad spectrum of activity for the Cu(II)-MTSC complex.

#### 3.9.2. Antifungal Assessments of MTSC Ligand and Its Metal Complexes

Next, we have examined the antifungal activity of the MTSC ligand and its metal complexes against the *Aspergillus flavus* and *Candida albicans* fungi (Table 6). Our results revealed that, except for MTSC-Co^2+^ complexes, the MTSC ligand and its metal complexes possess a considerable antifungal activity against *Candida albicans* fungi. Contradictory to previous studies [53], the activity of MTSC ligand did not improve upon complexation with various metal complexes, except for mononuclear MTSC-Cu^2+^ complex **12,** which showed a comparable activity. Unexpectedly, all-metal complexes showed no activity against the *Aspergillus flavus*, while the MTSC ligand showed a moderate activity compared to the ketoconazole.

### 3.10. Molecular Docking

**Figure 4:**
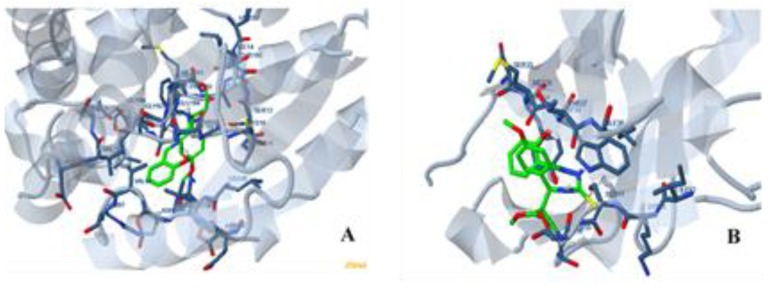
The 3D molecular docking of MTSC ligand against 3hb5-oxidoreductase protein (**A**) and speckle-type POZ protein (SPOP) protein binding (**B**).

To validate the biological activity of the MTSC ligand, we have performed in silico molecular docking study using Docking Server [34,35,36,37] to investigate the binding interaction of the MTSC ligand with the 3hb5-oxidoreductase breast cancer protein and the SPOP protein binding of kidney cancer (Figure 4A,B). The molecular docking results are shown in Table 7. Molecular docking is a significant method in computational drug design [34]. The principle point in molecular docking is to assume the molecular detection procedure. Molecular docking plans to accomplish the maximized confirmation for both the protein and the ligand with relative orientation between both of them.

The first results of molecular docking were examined by calculating the affinity energy of the MTSC ligand toward 3hb5-oxidoreductase protein and SPOP binding protein (Table 7). The MTSC free ligand showed a negative binding score (−5.83 kcal mol^−1^) toward 3hb5-oxidoreductase protein, which indicates that the interaction between the MTSC ligand and the 3hb5-oxidoreductase protein is thermodynamically favorable. Consequently, the decrease in the affinity energy will increase the binding affinity of the MTSC ligand toward the 3hb5-oxidoreductase protein active sites and will help to increase the inhibitory effect of the MTSC ligand. It was useful to locate the hydrogen-bonding interaction between the amino acids of the 3hb5-oxidoreductase protein with the MTSC ligand to evaluate the stability of the MTSC ligand. As shown in Figure 4A, the MTSC ligand showed the ability to fit and bind to the cavity of the 3hb5-oxidoreductase protein through several hydrogen-bonding interactions. It could be speculated that these hydrogen-bonding interactions may help in stabilizing the binding between MTSC and the 3hb5-oxidoreductase protein (Figure 4A). Additionally, the MTSC showed a high intermolecular binding energy value (−9.27 kcal mol^−1^) to the 3hb5-oxidoreductase protein (Table 7). These results confirm that the MTSC ligand is an effective inhibitor against the 3hb5-oxidoreductase protein. On the other hand, the high-affinity energy of the MTSC ligand toward SPOP binding protein (312.12 kcal/mol) indicates a low inhibitory effect of the MTSC against SPOP protein (Table 7). Overall, the molecular docking results revealed that MTSC can bind to the 3hb5-oxidoreductase protein and may exhibit a significant regulation on this target protein rather than SPOP binding protein. Our results are in agreement with several reports [54,55] that found the Schiff base ligand is an efficient inhibitor toward 3hb5-oxidoreductase breast cancer protein rather than SPOP kidney cancer binding protein.

## 4. Conclusion

In this study, we have synthesized a new methoxy thiosemicarbazone derivative (MTSC) as a potential ligand for metal complexation. The MTSC ligand was used to synthesize a set of eight new mononuclear and binuclear Co^2+^, Ni^2+^, Cu^2+^, and Zn^2+^ metal complexes. The structure of synthesized metal complexes was fully characterized by several analytical tools, including elemental analysis, molar conductivity, spectroscopic techniques (mass, NMR, UV-Vis, FTIR), and thermogravimetric analysis. Evaluation of the biological activity revealed that the MTSC ligand and its metal complexes possess considerable antibacterial activity. The molecular docking study revealed that the MTSC ligand could be a potential candidate inhibitor for the oxidoreductase protein.

## Figures and Tables

**Table 1:** Data of MTSC and its complexes.

Compounds	Color	%Found (% Calcd.)					
		C%	H%	N%	Cl%	M%	S%
**MTSC**	Yellow	58.03(58.14)	4.61(4.88)	12.12(12.33)	-	-	7.39(7.06)
**I**	Brown	38.11(38.29)	5.59(5.70)	12.09(12.18)	5.09(5.14)	8.43(8.54)	4.60(4.65)
**II**	Dark brown	30.21(30.35)	5.80(5.91)	14.31(14.48)	8.10(8.15)	13.43(13.54)	3.61(3.68)
**III**	Orange brown	40.33(40.42)	5.23(5.40)	12.77(12.85)	5.33(5.42)	8.78(8.98)	4.88(4.90)
**IV**	Reddish brown	33.09(33.11)	5.30(5.43)	15.76(15.80)	8.81(8.89)	14.65(14.71)	3.89(4.02)
**V**	Brown	39.01(39.05)	5.45(5.51)	12.33(12.42)	5.19(5.24)	9.29(9.39)	4.70(4.74)
**VI**	Green	32.44(32.56)	4.80(4.84)	8.56(8.63)	8.65(8.74)	15.56(15.66)	3.91(3.95)
**VII**	Yellow	42.09(42.18)	4.45(4.67)	8.91(8.94)	5.60(5.66)	10.34(10.44)	5.10(5.12)
**VIII**	Yellow	35.23(35.55)	4.11(4.20)	9.33(9.42)	9.41(9.54)	17.54(17.60)	4.22(4.31)

**Table 2:** IR frequency values (cm^−1^) for MTSC (free ligand) and its metal complexes.

Compounds	*ν*(O-H)	*ν*(N-H)	*ν*(COCH_3_)	*ν*(C=N)	*ν_as_*(COO)	*ν_s_*(COO)	*ν*(C–O)	*ν*(C=S)	*ν*(M–O)	*ν*(M–N)
**MTSC**	3340	-	2843	1589	-	-	1211	1018	-	-
**I**	3313	3174	2835	1508	1600	1438	1172	968	551, 516	443, 420
**II**	3332	3170	2835	1512	1589	1435	1180	1018	555, 516	443, 421
**III**	3344	3190	2835	1531	1604	1454	1172	972	594, 570	455, 432
**IV**	3317	3232	2831	1527	1604	1458	1176	968	594, 524	447, 416
**V**	3321	3194	2835	1504	1604	1454	1172	968	586, 516	470, 439
**VI**	3336	3012	2860	1508	1604	1450	1172	972	558, 516	470, 447
**VII**	3309	3116	2897	1573	1600	1384	1161	1080	597, 551	455, 420
**VIII**	3236	3132	2831	1512	1612	1562	1172	964	516, 551	495, 430

**Table 3:** Thermogravimetric analysis data of MTSC and its metal complexes.

Complex	Steps	Temp Range/°C	DTG Peak/°C	Decomposed Assignments	Weight Loss Found (Calc.%)
**MTSC**	1st	20–295	195 495	10C_2_H_2_ + CO + N_2_	69(69.7)
	2nd	295–530		2NO_2_	21(20)
	residue	530–800		H_2_S + carbon residue	10(10.1)
**8**	1st	35–203 203–460 460–620	60 275 515	3H_2_O	8(7.8)
	2nd			3H_2_O + 2H_2_ + 6C_2_H_2_ + 3NH_3_ + H_2_S + N_2_ + ½Cl_2_	52(52.5)
	3rd			CO_2_ + NO_2_	13(13.1)
	residue			CoO + carbon residue	27(26.4)
**9**	1st	35–180	52 130,220 450	6H_2_O	13(12.4)
	2nd	180–420		H_2_ + 4NH_3_ + 9C_2_H_2_ + 4CO + H_2_S	52(51.7)
	3rd	420–547		Cl_2_ + 2N_2_ + NH_3_ + H_2_O	17(18.5)
	residue	547–800		2CoO	17(17.2)
**10**	1st	35–249	45 300 445	2H_2_O	5(5.5)
	2nd	249–370		3NH_3_ + H_2_O + ½Cl_2_ + H_2_S + 4H_2_	23(22.4)
	3rd	370–585		H_2_O + N_2_ + NO + 3CO + 4C_2_H_2_	40.5(40.5)
	residue	585–800		NiO + carbon residue	31.5(31.4)
**11**	1st	25–230	145 296 445	2H_2_O + 3NH_3_	11(10.9)
	2nd	230–375		2NH_3_ + Cl_2_ + H_2_S + 4H_2_	19(18.5)
	3rd	375–551		5C_2_H_2_ + 2NO + H_2_O + N_2_	34(33.9)
	residue	551–800		2NiO + carbon residue	36(36.7)
**12**	1st	20–175	90 210,255,330 504 650	3H_2_O	8(8)
	2nd	175–405		3NH_3_ + 5C_2_H_4_	27(26.76)
	3rd	405–605		2H_2_O + 2NH_3_ + H_2_S + NO_2_ + ½Cl_2_	28(27.54)
	4th	605–710		CO_2_	6(6.5)
	residue	710–800		CuO+ carbon residue	31(31.6)
**13**	1st	20–155	40 215,305,359 655	2H_2_O	4(4.4)
	2nd	155–525		6H_2_O + NH_3_ + Cl_2_ + H_2_S + N_2_ + NO + 9H_2_	38(37.8)
	3rd	525–740		NO_2_	6(5.7)
	residue	740–800		2CuO + carbon residue	52(52.4)
**14**	1st	30–180	120,140 255 530,570	H_2_O	3(2.9)
	2nd	180–450		H_2_O + NO_2_ + 6C_2_H_2_	35(35.1)
	3rd	450–630		½Cl_2_ + NH_3_ + N_2_ + H_2_S + H_2_O + H_2_ + CO_2_	30(32)
	residue	630–800		ZnO + carbon residue	31.5(30)
**15**	1st	25–345	160,245 540	2H_2_O + 2NO_2_ + N_2_ + 7C_2_H_2_	46(45.5)
	2nd	345–545		Cl_2_ + H_2_S + CH_4_ + H_2_O + H_2_ + NH_3_	21(21.4)
	residue	545–800		2ZnO + carbon residue	33(33.1)

**Table 4:** The Coats–Redfern (CR) and Horowitz–Metzger (HM) calculations to the MTSC and its metal complexes.

Compound	Stage	Method	Parameter					r
			E*	A	ΔS	ΔH	ΔG	
			(J mol^−1^)	(s^−1^)	(J mol^−1^ K^−1^)	(J mol^−1^)	(J mol^−1^)	
**MTSC**	1st 2nd	CR	3.32 × 10^−1^	5.15 × 10^−9^	−4.07 × 10^2^	−3.77 × 10^3^	1.81 × 10^5^	0.9026
		HM	7.96 × 10^2^	9.60 × 10^−5^	−3.25 × 10^2^	−2.97 × 10^3^	1.44 × 10^5^	0.9205
		CR	9.31 × 10^−2^	1.40 × 10^−9^	−4.21 × 10^2^	−5.76 × 10^3^	2.86 × 10^5^	0.991
		HM	1.52 × 10^3^	8.28 × 10^−5^	−3.30 × 10^2^	−4.24 × 10^3^	2.25 × 10^5^	0.983
**8**	1st 2nd	CR	1.17 × 10^−1^	2.23 × 10^−9^	−4.15 × 10^2^	−4.27 × 10^3^	2.09 × 10^5^	0.9965
		HM	1.01 × 10^3^	9.79 × 10^−5^	−3.26 × 10^2^	−3.25 × 10^3^	1.64 × 10^5^	0.9272
		CR	1.56 × 10^−1^	4.41 × 10^−9^	−4.13 × 10^2^	−6.26 × 10^3^	3.04 × 10^5^	0.9939
		HM	1.52 × 10^3^	6.82 × 10^−5^	−3.32 × 10^2^	−4.75 × 10^3^	2.46 × 10^5^	0.996
**9**	1st 2nd	CR	1.97 × 10^−1^	6.93 × 10^−9^	−4.05 × 10^2^	−3.85 × 10^3^	1.84 × 10^5^	0.9939
		HM	8.51 × 10^2^	9.91 × 10^−5^	−3.25 × 10^2^	−3.00 × 10^3^	1.48 × 10^5^	0.9217
		CR	1.57 × 10^−1^	8.26 × 10^−9^	−4.07 × 10^2^	−6.05 × 10^3^	2.90 × 10^5^	0.9834
		HM	1.62 × 10^3^	7.97 × 10^−5^	−3.31 × 10^2^	−4.44 × 10^3^	2.36 × 10^5^	0.9877
**10**	1st 2nd	CR	4.57 × 10^−2^	2.10 × 10^−9^	−4.15 × 10^2^	−4.18 × 10^3^	2.05 × 10^5^	0.9113
		HM	6.45 × 10^2^	5.96 × 10^−5^	−3.30 × 10^2^	−3.54 × 10^3^	1.63 × 10^5^	0.9949
		CR	2.88 × 10^−1^	1.29 × 10^−8^	−4.04 × 10^2^	−6.30 × 10^3^	3.00 × 10^5^	0.9009
		HM	2.17 × 10^3^	1.07 × 10^−4^	−3.29 × 10^2^	−4.14 × 10^3^	2.45 × 10^5^	0.9868
**11**	1st 2nd 3rd	CR	2.44 × 10^−1^	1.35 × 10^−8^	−3.99 × 10^2^	−3.73 × 10^3^	1.75 × 10^5^	0.9798
		HM	5.90 × 10^2^	6.90 × 10^−5^	−3.28 × 10^2^	−3.14 × 10^3^	1.44 × 10^5^	0.9882
		CR	3.74 × 10^−1^	3.00 × 10^−9^	−4.14 × 10^2^	−4.77 × 10^3^	2.32 × 10^5^	0.9838
		HM	1.49 × 10^3^	1.24 × 10^−4^	−3.25 × 10^2^	−3.28 × 10^3^	1.83 × 10^5^	0.9715
		CR	3.88 × 10^−1^	1.16 × 10^−8^	−4.04 × 10^2^	−6.10 × 10^3^	2.90 × 10^5^	0.9814
		HM	1.72 × 10^3^	8.53 × 10^−5^	−3.30 × 10^2^	−4.37 × 10^3^	2.38 × 10^5^	0.9735
**12**	1st 2nd	CR	1.07 × 10^−1^	3.88 × 10^−9^	−4.10 × 10^2^	−4.14 × 10^3^	2.00 × 10^5^	0.9756
		HM	8.29 × 10^2^	8.18 × 10^−5^	−3.27 × 10^2^	−3.31 × 10^3^	1.60 × 10^5^	0.9654
		CR	1.47 × 10^−1^	5.18 × 10^−9^	−4.12 × 10^2^	−6.64 × 10^3^	3.22 × 10^5^	0.9118
		HM	1.90 × 10^3^	7.96 × 10^−5^	−3.32 × 10^2^	−4.74 × 10^3^	2.60 × 10^5^	0.9936
**13**	1st 2nd	CR	1.71 × 10^−1^	4.42 × 10^−9^	−4.08 × 10^2^	−3.73 × 10^3^	1.79 × 10^5^	0.9883
		HM	7.71 × 10^2^	9.46 × 10^−5^	−3.25 × 10^2^	−2.96 × 10^3^	1.43 × 10^5^	0.956
		CR	1.41 × 10^−1^	1.02 × 10^−8^	−4.05 × 10^2^	−5.68 × 10^3^	2.71 × 10^5^	0.9632
		HM	1.28 × 10^3^	6.87 × 10^−5^	−3.32 × 10^2^	−4.40 × 10^3^	2.22 × 10^5^	0.9936
**14**	1st 2nd	CR	1.17 × 10^−1^	2.90 × 10^−9^	−4.14 × 10^2^	−4.60 × 10^3^	2.24 × 10^5^	0.9798
		HM	9.65 × 10^2^	7.80 × 10^−5^	−3.29 × 10^2^	−3.63 × 10^3^	1.78 × 10^5^	0.9709
		CR	1.32 × 10^−1^	3.59 × 10^−9^	−4.15 × 10^2^	−7.09 × 10^3^	3.47 × 10^5^	0.9987
		HM	2.67 × 10^3^	1.07 × 10^−4^	−3.30 × 10^2^	−4.43 × 10^3^	2.77 × 10^5^	0.9782
**15**	1st 2nd	CR	5.70 × 10^−1^	8.61 × 10^−9^	−4.02 × 10^2^	−3.60 × 10^3^	1.71 × 10^5^	0.9109
		HM	9.65 × 10^2^	7.80 × 10^−5^	−3.29 × 10^2^	−3.63 × 10^3^	1.78 × 10^5^	0.9994
		CR	5.15 × 10^−2^	1.51 × 10^−9^	−4.20 × 10^2^	−5.51 × 10^3^	2.73 × 10^5^	0.9546
		HM	1.24 × 10^3^	7.10 × 10^−5^	−3.31 × 10^2^	−4.27 × 10^3^	2.15 × 10^5^	0.9895

**Table 5:** Antibacterial assessment (inhibition zone diameter) of MTSC ligand and its metal complexes at a concentration of 1 μM.

Ligand/Complex	Gram-Positive Bacteria		Gram-Negative Bacteria	
	*Bacillus* *subtilis*	*Staphylococcus aureus*	*E. coli*	*Proteus vulgaris*
**MTSC**	15 ± 1.2 ^d^	NA	10 ± 0.7 ^e^	9 ± 0.3 ^e^
**8**	15 ± 0.9 ^d^	10 ± 0.8 ^d^	13 ± 0.8 ^d^	14 ± 0.7 ^c,d^
**9**	16 ± 1.1 ^d^	12 ± 0.9 ^d^	16 ± 0.9 ^c^	15 ± 0.5 ^c^
**10**	21 ± 0.7 ^c^	15 ± 1.1 ^c^	19 ± 1.1 ^b^	15 ± 0.8 ^c^
**11**	22 ± 0.9 ^c^	18 ± 1.3 ^b^	21 ± 1.2 ^b^	19 ± 1.1 ^b^
**12**	33 ± 1.8 ^a^	18 ± 0.8 ^b^	22 ± 1.1 ^b^	25 ± 1.2 ^a^
**13**	33 ± 1.2 ^a^	17 ± 1.2 ^b^	20 ± 0.9 ^b^	26 ± 1.5 ^a^
**14**	17 ± 1.3 ^d^	12 ± 0.8 ^d^	11 ± 0.3 ^e^	12 ± 0.8 ^d^
**15**	21 ± 0.5 ^c^	14 ± 0.4 ^c^	16 ± 0.6 ^c^	14 ± 0.4 ^c,d^
Gentamicin	26 ± 1.7 ^b^	24 ± 1.8 ^a^	30 ± 2.3 ^a^	25 ± 1.9 ^a^

Each value is the mean of three experiments ± SEM. Different superscript letters designate significant differences (*p <* 0.05) using Duncan’s multiple range test.

**Table 6:** Antifungal activities of MTSC ligand and its metal complexes (inhibition zone diameter) at a concentration of 1 μM.

Ligand/Complex	*Aspergillus flavus*	*Candida albicans*
**MTSC**	10 ± 0.4 ^b^	11 ± 0.8 ^b^
**8**	NA	NA
**9**	NA	NA
**10**	NA	9 ± 0.4 ^c^
**11**	NA	8 ± 0.2 ^c^
**12**	NA	12 ± 0.7 ^b^
**13**	NA	8 ± 0.3 ^c^
**14**	NA	8 ± 0.2 ^c^
**15**	NA	9 ± 0.3 ^c^
Ketoconazole	16 ± 0.7 ^a^	20 ± 1.1 ^a^

Each value is the mean of three experiments ± SEM. Different superscript letters designate significant differences (*p <* 0.05) using Duncan’s multiple range test.

**Table 7:** Docking scores of the MTSC ligand in the oxidoreductase protein and SPOP binding protein sites.

	Est. Free Energy of Binding	Est. Inhibition Constant, Ki	vdW+Hbond+Desolv Energy	Electrostatic Energy	Total Intermolec. Energy	Interact. Surface
Oxidoreductase protein	−5.83 kcal/mol	53.71 μM	−8.96 kcal/mol	−0.30 kcal/mol	−9.27 kcal/mol	1005.512
SPOP protein	312.12 kcal/mol	-	231.33 kcal/mol	−0.15 kcal/mol	231.18 kcal/mol	543.026

## Data Availability

All data are provided in the manuscript.

## Supplementary Materials

The following are available online, Figure S1: ^1^H-NMR spectrum of the MTSC ligand in DMSO-d_6_ solvent, Figure S2: ^1^H-NMR spectrum of the [Zn(MTSC)(NH_3_)(Cl)].3H_2_O (**14**) in DMSO-d_6_ solvent, Figure S3: ^1^H-NMR spectrum of the [Zn_2_(MTSC)(NH_3_)_3_(Cl)_2_].2H_2_O (**15**) in DMSO-d_6_ solvent, Figure S4: TG curves of Co(II) complexes **8** (a) and **9** (b), Figure S5: TG curves of Ni(II) complexes **10** (a) and **11** (b), Figure S6: TG curves of Cu(II) complexes **12** (a) and **13** (b), Figure S7: TG curves of Zn(II) complexes **14** (a) and **15** (b).
