# Supplementary material for: New Mononuclear and Binuclear Cu(II), Co(II), Ni(II), and Zn(II) Thiosemicarbazone Complexes with Potential Biological Activity: Antimicrobial and Molecular Docking Study
Source: Molecules. 2021 Apr 15;26(8):2288. doi: 10.3390/molecules26082288 (PMC8071257; doi:10.3390/molecules26082288)
Supplement: Supplementary file 1 [file molecules-26-02288-s001.zip › molecules-1156867-supplementary.pdf]

molecules

New Mononuclear and Binuclear Cu(II), Co(II), Ni(II), and Zn(II) Thiosemicarbazone Complexes with Potential Biological Activity: Antimicrobial and Molecular Docking Study

Ahmed Gaber [1,2], Moamen S. Refat [3,*], Arafa A.M. Belal [4], Ibrahim M. El-Deen [4], Nader Hassan [4], Rozan Zakaria [4], Majid Alhomrani [2,5], Abdulhakeem S. Alamri [2,5], Walaa F. Alsanie [2,5] and Essa M. Saied [6,7]

1 Department of Biology, College of Science, Taif University, P.O. Box 11099, Taif 21944, Saudi Arabia
2 Center of Biomedical Sciences Research, Taif University, P.O. Box 11099, Taif 21944, Saudi Arabia
3 Department of Chemistry, College of Science, Taif University, P.O. Box 11099, Taif 21944, Saudi Arabia
4 Department of Chemistry, Faculty of Science, Port Said University, Port Said 42511, Egypt
5 Department of Clinical Laboratory Sciences, College of Applied Medical Sciences, Taif University, P.O. Box 11099, Taif 21944, Saudi Arabia
6 Chemistry Department, Faculty of Science, Suez Canal University, P.O. Box 41522, Ismailia 42524, Egypt
7 Institute for Chemistry, Humboldt Universität zu Berlin, Brook-Taylor-Str. 2, 12489 Berlin, Germany

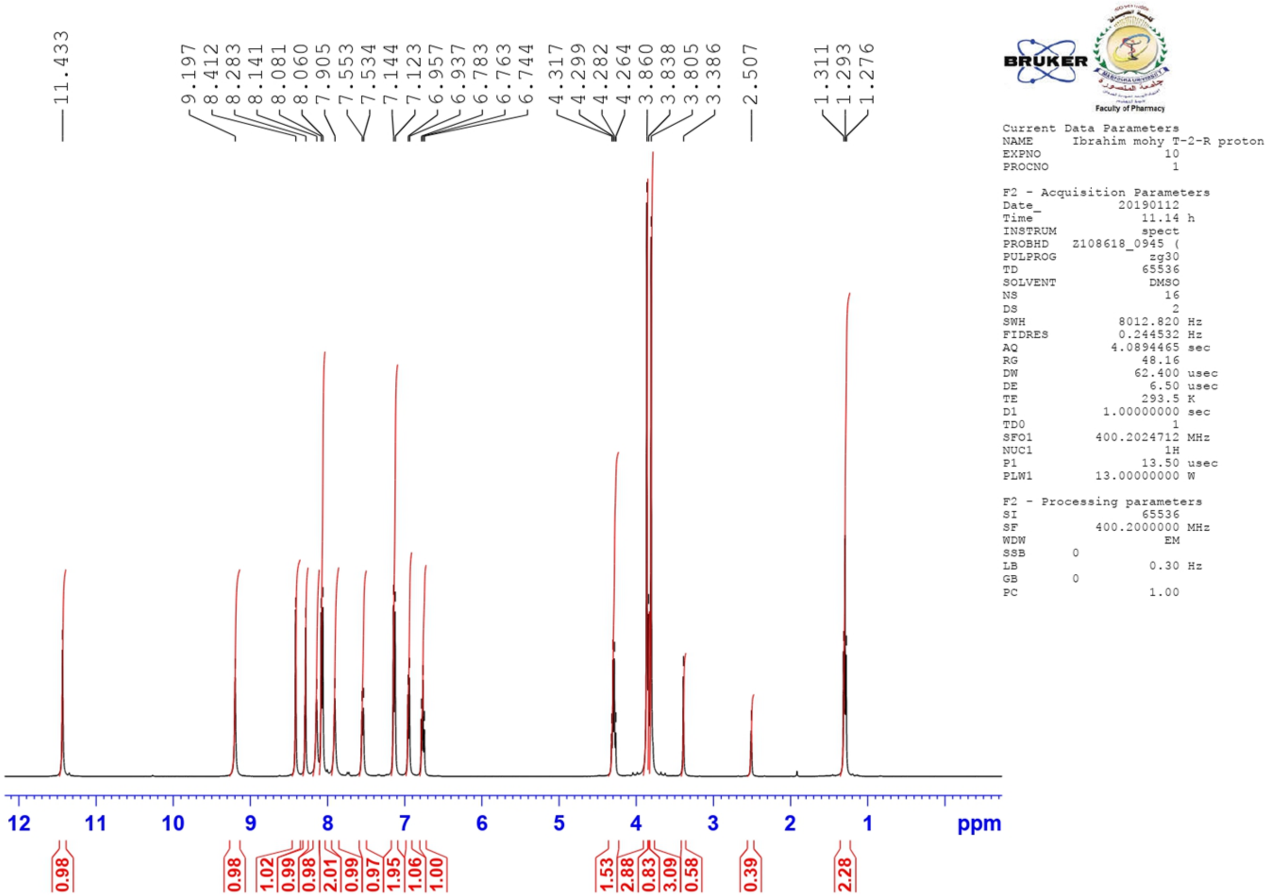

Figure S1: ^{1}H-NMR spectrum of the MTSC ligand in DMSO-d_6 solvent.

molecules

MDPI

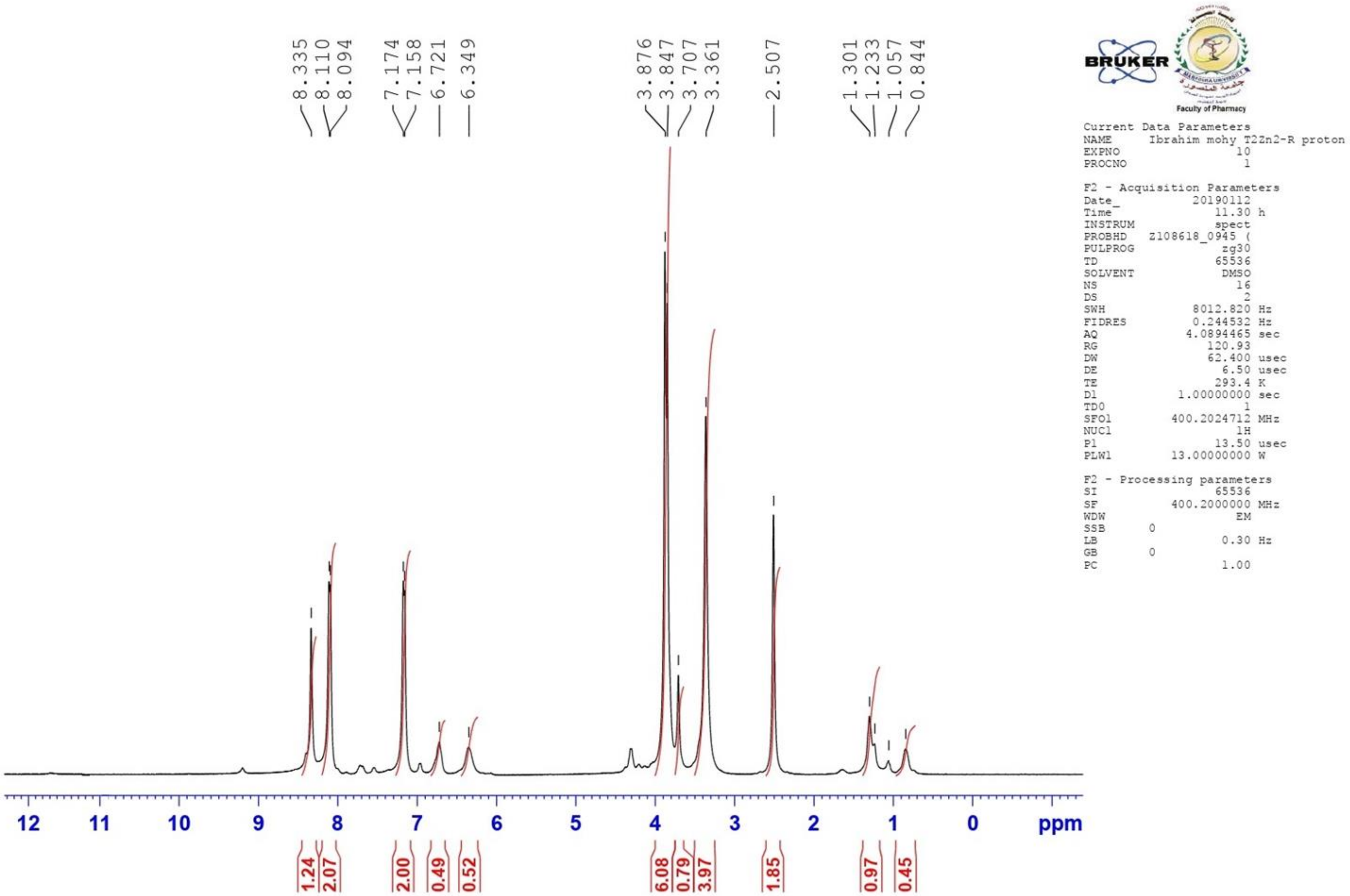

Figure S2: ^{1}H-NMR spectrum of the $[Zn(MTSC)(NH_3)(Cl)].3H_2O$ (**14**) in DMSO-d_6 solvent.

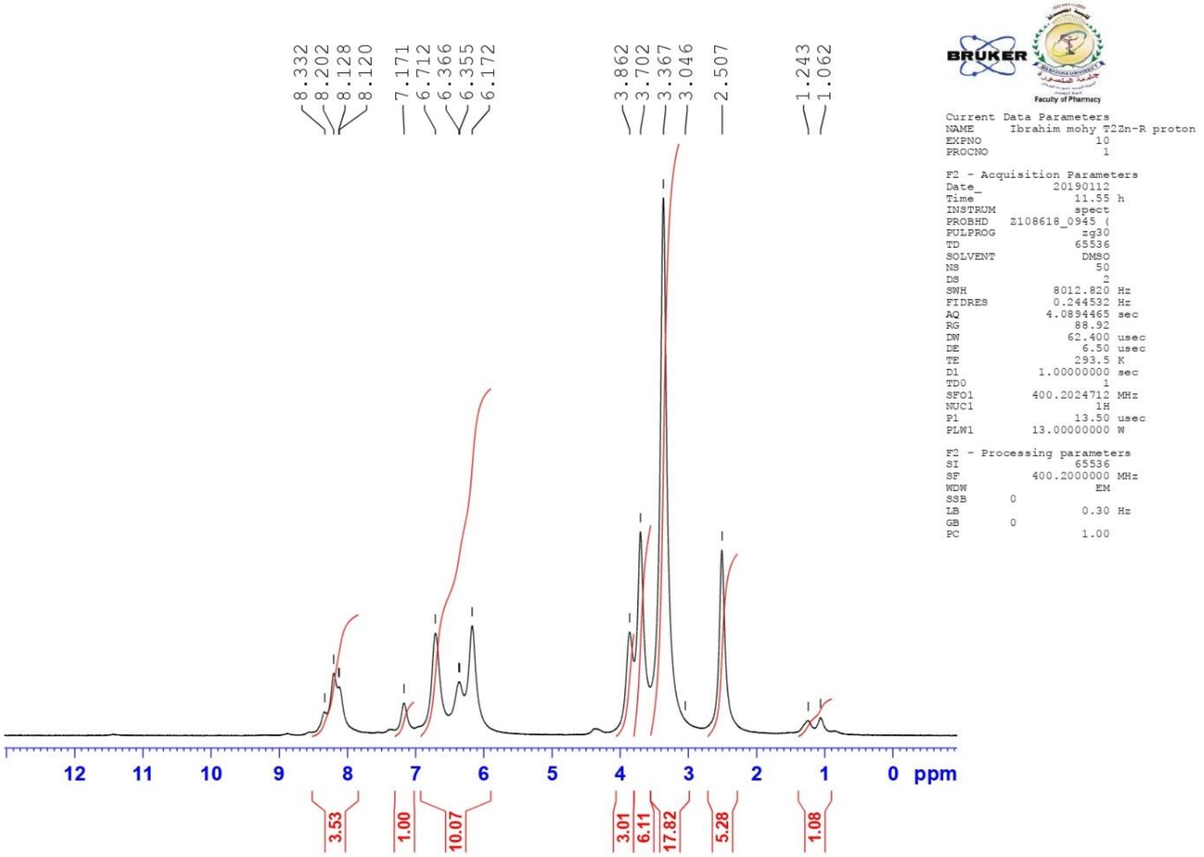

Figure S3: ^{1}H-NMR spectrum of the $[Zn_2(MTSC)(NH_3)_3(Cl)_2].2H_2O$ (**15**) in DMSO-d_6 solvent.

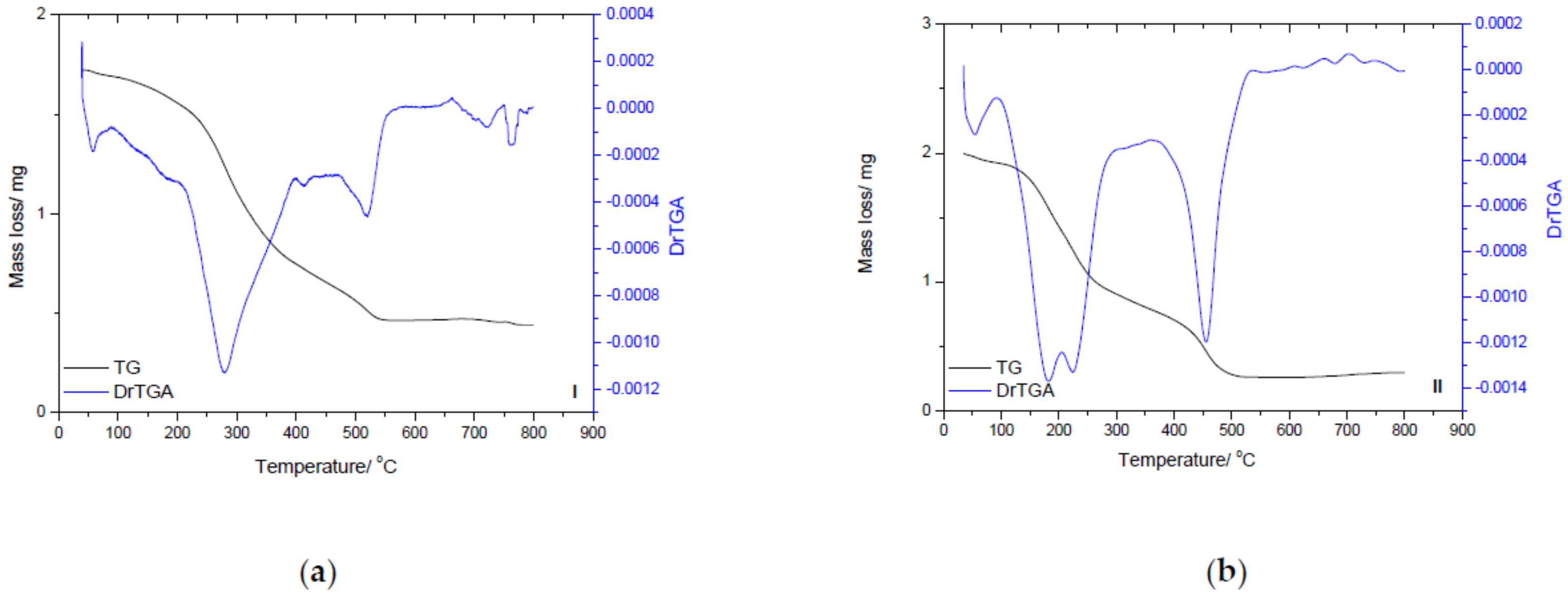

(a) (b)

Figure S4: TG curves of Co(II) complexes **8** (a) and **9** (b).

molecules

MDPI

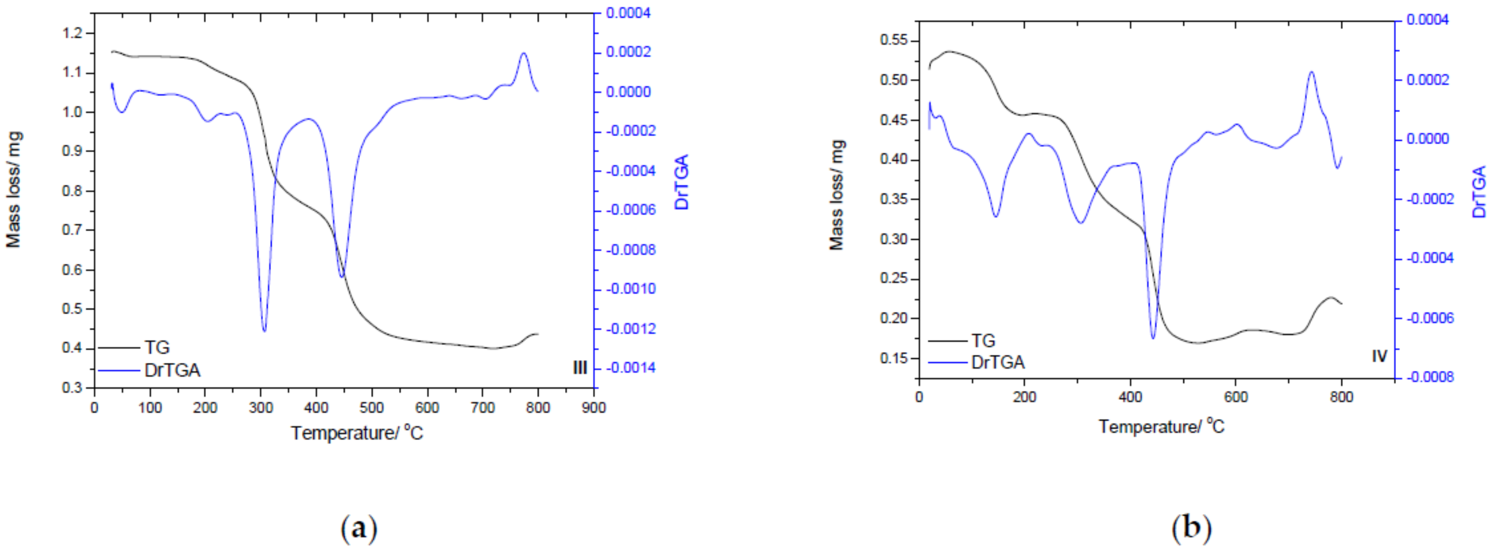

Figure S5: TG curves of Ni(II) complexes **10** (a) and **11** (b).

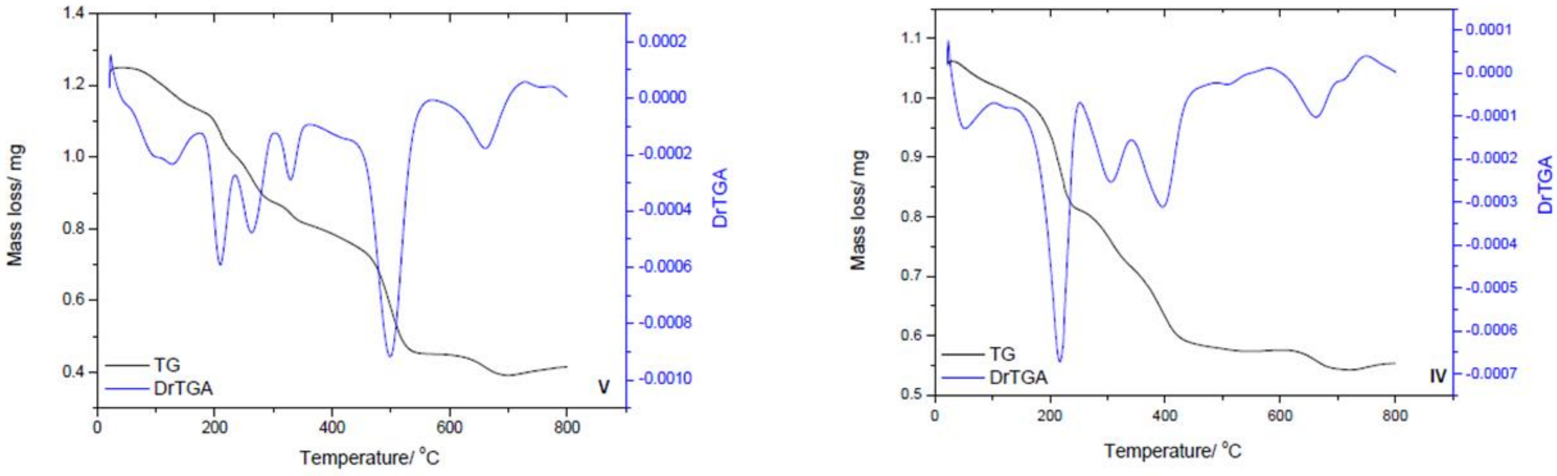

(a) (b)

Figure S6: TG curves of Cu(II) complexes **12** (a) and **13** (b).

molecules

MDPI

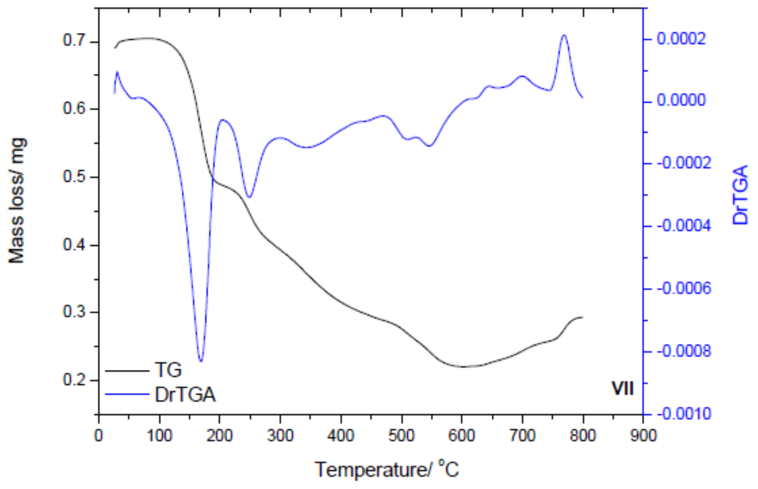

(a)

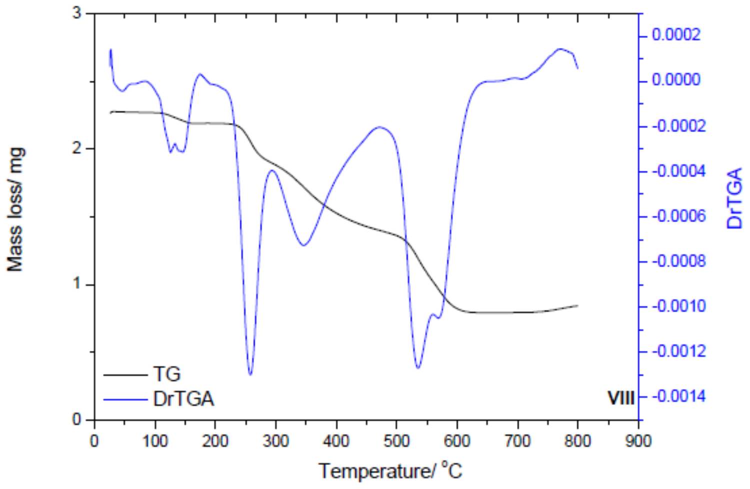

(b)

Figure S7: TG curves of Zn(II) complexes **14** (a) and **15** (b).
